# Supplementary material for: Safranal Induces Vasorelaxation by Inhibiting Ca2+ Influx and Na+/Ca2+ Exchanger in Isolated Rat Aortic Rings
Source: Molecules. 2022 Jun 30;27(13):4228. doi: 10.3390/molecules27134228 (PMC9268204; doi:10.3390/molecules27134228)
Supplement: Supplementary file 1 [file molecules-27-04228-s001.zip › molecules-1711316-supplementary.pdf]

Supplementary Figures

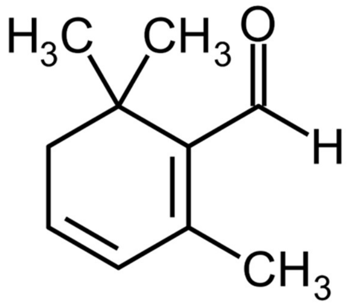

Figure S1. The chemical structure of safranal.

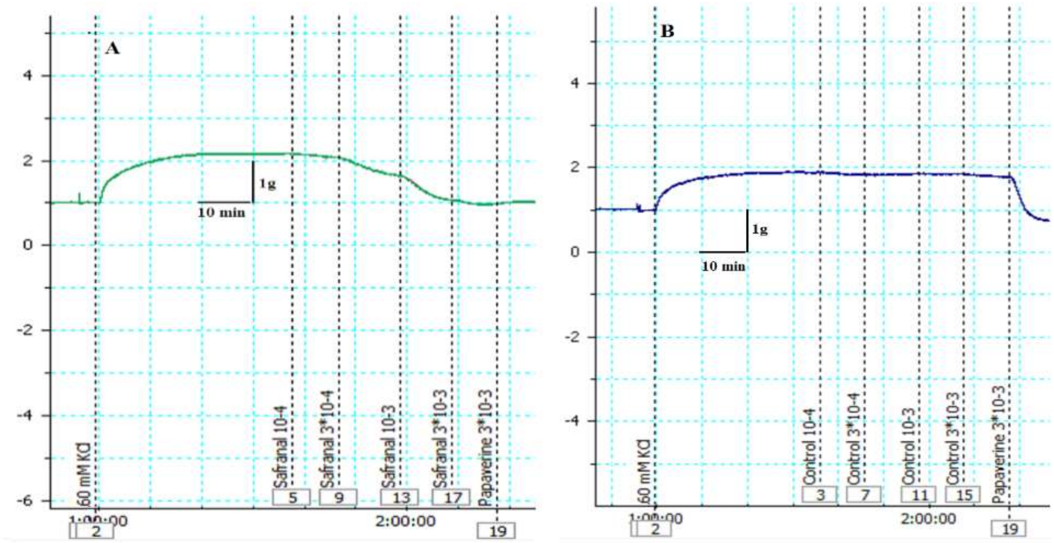

Figure S2. A. Concentration-response curve of safranal vasodilation in the indicated concentrations of aorta precontracted with 60mM KCl. **B.** The lack of effect for the solvent of safranal. Papaverine was used at the end of the experiment to induce maximum relaxation.

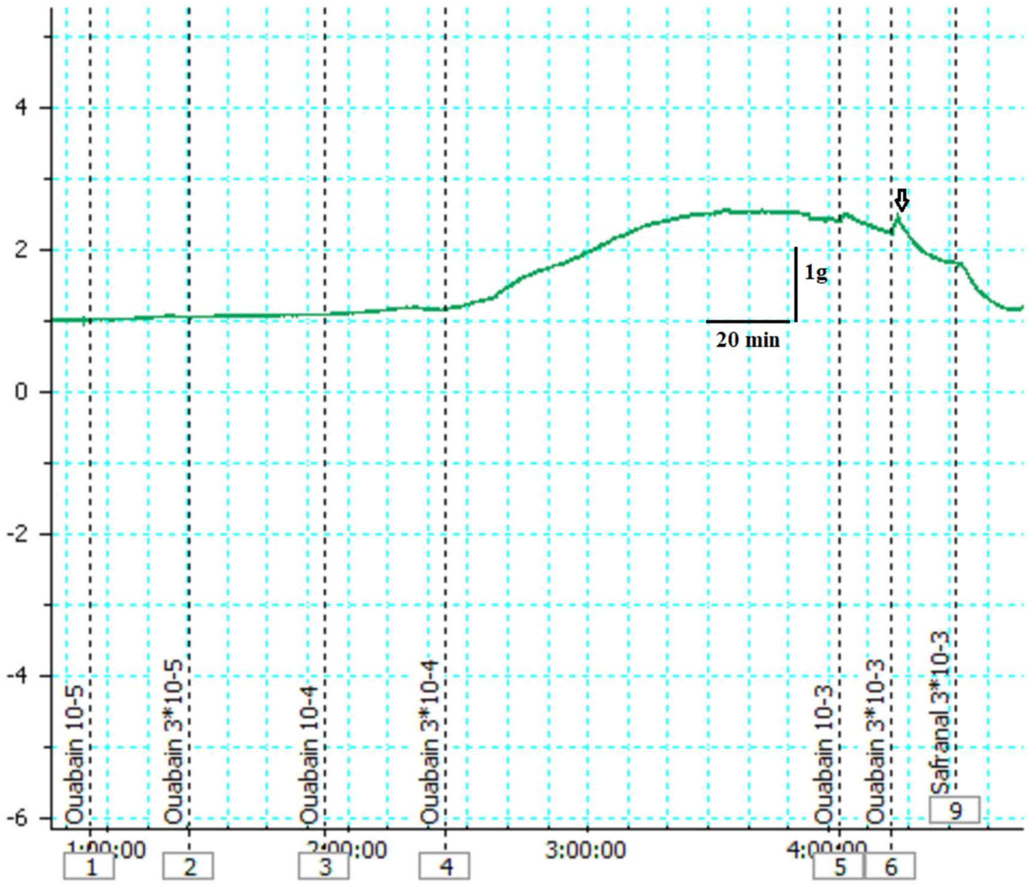

Figure S3. Concentration-effect curve of ouabain (10^{-5}-$3x10^{-3}$M). This curve shows that small concentrations of ouabain are relatively ineffective in causing the Na/Ca exchanger to work in reverse mode and causes a contraction. Only when 3x 10^{-4}M was used we obtained a significant tonic contraction. Most interesting is that larger concentration, especially 3x 10^{-3}M which has been reported to block completely Na/K pump population, was found to cause a phasic contraction (arrow) that was decaying quickly (3–4 minutes), and was followed by a relaxation. This response could be due to depolarization due to K^+ accumulation extracellularly (Reference 42) or due to a burst of reactive oxygen species as reported by Pongrakhananon V et al; PLoS One 8 (7): e68623 (2013). https://www.ncbi.nlm.nih.gov/pmc/ articles/PMC3707866.
